# High-Resolution CT Findings as Predictive Factors for Recurrent Nontuberculous Mycobacterial Pulmonary Disease after Successful Treatment

**DOI:** 10.3390/jcm10020172

**Published:** 2021-01-06

**Authors:** Hyewon Choi, Min Jae Cha, Yang Soo Kim, Jae Chol Choi

**Affiliations:** 1 Department of Radiology, Chung-Ang University Hospital, Chung-Ang University College of Medicine, 102 Heukseok-ro, Dongjak-gu, Seoul 06973, Korea; hyewon101@hotmail.com (H.C.); asterism35@naver.com (M.J.C.); ysjy007@cau.ac.kr (Y.S.K.); 2 Division of Pulmonary Medicine, Department of Internal Medicine, Chung-Ang University, Chung-Ang University College of Medicine, 102 Heukseok-ro, Dongjak-gu, Seoul 06973, Korea

**Keywords:** nontuberculous mycobacteria, recurrence, computed tomography

## Abstract

Despite long-term treatment for nontuberculous mycobacterial pulmonary disease (NTM-PD), recurrence is common. We aim to identify computed tomography (CT) findings that predict recurrence after successful treatment of NTM-PD. This retrospective study included 44 patients (12 men, 60 ± 11.2 years) successfully treated for NTM-PD between March 2009 and September 2016. Recurrence developed in 18 patients (40.9%) during follow-up (median, 852 days). CT scores for bronchiectasis, bronchiolitis, consolidation, cavities, and nodules at the initiation and termination of treatment were evaluated, then determined association with recurrence. We also assessed the diagnostic performance and reproducibility of CT scores. Patients with recurrent NTM-PD showed higher CT scores for bronchiectasis (*p* = 0.008), nodules (*p* = 0.006), consolidation (*p* = 0.033), and total CT scores (*p* = 0.017) at the time of treatment termination. On the contrary, only nodule score differed among the initial CT scores (*p* = 0.014). Regression analysis showed that the scores for bronchiectasis (odds ratio (OR) = 1.638, 95% confidence interval (CI) = 1.049–2.558, *p* = 0.030) and nodules (OR = 5.246, 95% CI = 1.370–20.087, *p* = 0.016) at treatment termination were significant predictors. The AUC of the regression model was 0.814 (95% CI = 0.689–0.939, *p* = 0.005). The interreader agreement for the total CT score was excellent (intraclass correlation coefficient = 0.841, *p* < 0.001). CT scores at the time of treatment termination can predict disease recurrence with good reproducibility.

## 1. Introduction

The incidence and prevalence of nontuberculous mycobacteria pulmonary diseases (NTM-PD) have been increasing worldwide [1]. In the United States, the annual incidence and prevalence of NTM-PD were estimated to increase by 5.2% and 7.5% from 2008 to 2015, respectively [2]. Clinical, radiological, and microbiological criteria are equally critical for the diagnosis of NTM-PD [3]. The most common species that induced disease is *Mycobacterium avium* complex (MAC), followed by *Mycobacterium abscessus* complex. The characteristic computed tomography (CT) findings of NTM-PD are bilateral bronchiectasis, multifocal bronchiolitis, lobular consolidation, and cavitation [4].

The mainstay treatment for NTM-PD is the long-term use of multiple antibiotics [5]. However, the treatment outcome has been mostly unsatisfactory. Overall treatment success was recorded in only 40–60% of patients, and microbiological recurrence is reported in 30–50% of the patients despite successful treatment [6]. Therefore, it is crucial to determine the predictive factors associated with recurrent disease following successful treatment.

Chest CT has been used in a wide variety of clinical settings for disease monitoring, determination of the timing of treatment initiation, and evaluation of the treatment response in NTM-PD [7,8,9]. Indeed, various studies have reported the radiologic course of MAC-pulmonary disease (MAC-PD) and *M. massiliense* infection [7,8,9,10,11]. Previous studies also reported a nodular bronchiectatic form on baseline CT was an independent predictor of recurrence in MAC-PD [6,12].

We considered that quantitative chest CT findings could be further substantiated as imaging biomarkers for predicting NTM-PD recurrence and hypothesized that a larger extent of lung abnormalities at the time of treatment termination could be predictive of the higher rates of microbiological recurrence even after successful treatment. Thus, this study aimed to investigate the predictive imaging parameters of chest CT for the recurrence of NTM-PD following successful treatment.

## 2. Materials and Methods

The institutional review board of Chung-Ang University Hospital approved this retrospective study (IRB number: 1811-011-16224) and the need for written informed consent was waived.

### 2.1. Study Population

**Figure 1:**
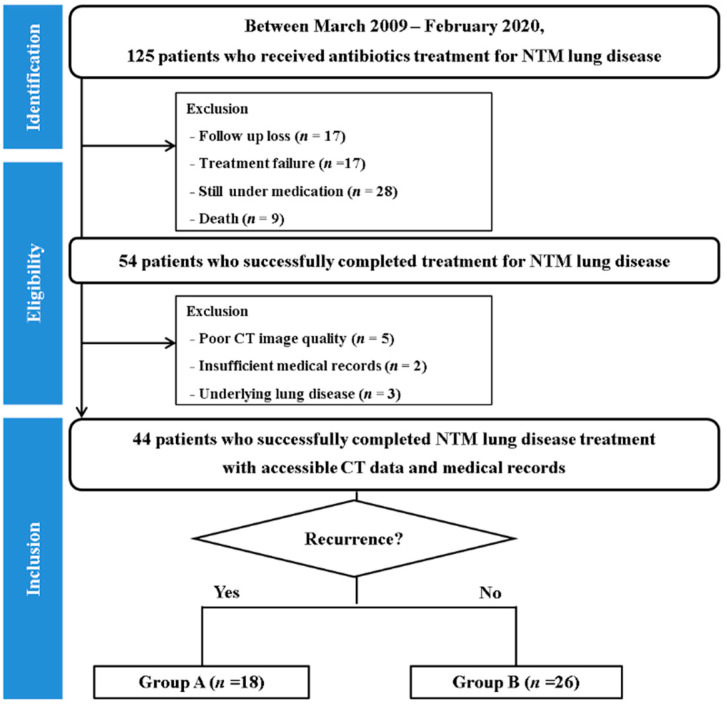
Flowchart of patient enrolment.

Between March 2009 and September 2016, 52 patients who were successfully treated for NTM-PD and had undergone serial chest CT at the time of initiation and termination of treatment in our institution were identified. The patients met the diagnostic criteria for NTM-PD based on the American Thoracic Society guidelines [3]. Treatment success was defined based on the absence of a positive culture result for at least 12 months of continuous treatment following sputum conversion. Eight patients were excluded owing to poor CT image quality (*n* = 6) and the presence of concomitant lung disease (*n* = 2). Finally, 44 patients (mean age, 60.2 ± 11.2 years; male-to-female sex ratio, 12:32) were included, grouped into patients with recurrence (*n* = 18) and non-recurrence (*n* = 26) during follow-up (median, 852 days; interquartile range (IQR), 489.5–1917.5 days) (Figure 1). Microbiological recurrence was defined as at least two positive cultures of the causative species from respiratory samples after the cessation of anti-mycobacterial treatment [13]. Data on clinical factors, including age, sex, body mass index (BMI), medical history, and duration of NTM-PD treatment were collected from the electronic medical record system.

### 2.2. CT Acquisition

CT scans were obtained using a 64-detector row (Brilliance; Philips Healthcare, Cleveland, OH, USA), 128-detector row (Optima 660; GE Healthcare, Waukesha, WI, USA), or 256-detector row (iCT; Philips Healthcare) CT scanner. Scans were obtained from the lung apices to the lung bases. The CT acquisition parameters were as follows: 120–140 kVp; 100–200 mA; reconstruction interval, 1–2.5 mm; and section thickness, 1.3–2.5 mm and 1–2.5 mm for axial and coronal images, respectively. All CT data were reconstructed using a high spatial frequency algorithm.

### 2.3. CT Interpretation

Two thoracic radiologists (H.C. and M.J.C. with 5 and 9 years of experience, respectively) who were blinded to the clinical data independently reviewed all CT images randomly. Initial CT abnormalities were classified based on distinct patterns: (i) fibrocavitary forms, (ii) nodular bronchiectatic forms, and (iii) unclassifiable forms (when the CT did not detect any specific pattern and showed multifocal consolidation and multiple nodules) [8,14]. The CT scans obtained at both the time of treatment initiation and the time of treatment termination were analyzed for scoring. Six lobes (the lingular division of the left upper lobe was considered as a separate lobe) were assessed for the presence of lung parenchymal abnormalities, including bronchiectasis (when the bronchial lumen diameter was greater than the size of the adjacent pulmonary artery, in the absence of tapering of the bronchial lumen); cellular bronchiolitis (presence of small centrilobular nodules (<10 mm in diameter) and branching nodular structures); airspace consolidation (including all variants of distribution, including lobular, subsegmental, and segmental consolidations); and cavities [15]. These parenchymal abnormalities were scored according to a previously reported chest CT scoring system (Table S1) [7,8].

### 2.4. Statistical Analyses

Continuous data were expressed as means and standard deviations or medians with IQRs; categorical data were presented as proportions. The Mann–Whitney U test and Fisher’s exact test were performed to compare the baseline characteristics and CT scores between patients with and without recurrent NTM-PD. The CT scores obtained at the initiation and termination of treatment were compared using the Wilcoxon signed-rank test. Univariate and multivariate logistic regression analyses were performed to identify the predictors of recurrence. Additionally, receiver operating characteristic (ROC) curve analysis was performed for the prediction of NTM-PD recurrence using the total CT score and the probability of the regression model; areas under the curve (AUCs) were also assessed. Interobserver agreements for scores of the parenchymal abnormalities were assessed using intraclass correlation coefficients (ICCs). *p*-values < 0.05 were considered significant. All statistical analyses were performed using IBM SPSS Statistics for Windows version 25.0 (IBM Corp., Armonk, NY, USA).

## 3. Results

### 3.1. Baseline Characteristics

Recurrence was observed in 18 of the 44 NTM-PD patients. The median period from treatment termination to recurrence was 578 days (IQR, 333.5–1182 days). MAC was most frequently isolated in both the non-recurrent and recurrent NTM-PD groups, followed by *M. abscessus* complex. In the recurrent group, different species were isolated on recurrence in 10 patients (55.5%) (Table S2). There were no significant differences in age, BMI, treatment duration, and initial CT pattern between the two groups. The nodular bronchiectatic form was the most common in both the recurrent and non-recurrent groups (61.1% and 53.8%, respectively). Table 1 summarises the baseline characteristics of the two groups. Table S3 shows the treatment regimen according to NTM species and disease recurrence.

### 3.2. Comparison of CT Scores at the Time of Treatment Termination between Patients with and without NTM-PD Recurrence

CT scores obtained at the time of treatment initiation did not differ significantly between the two groups, except for the CT nodule score, which was significantly higher in the recurrent NTM-PD patients than in those without recurrence (1.17 ± 0.86 vs. 0.58 ± 0.51, *p* = 0.014). In contrast, the total CT score at the time of treatment termination was significantly higher in patients with NTM-PD recurrence than in those without NTM-PD recurrence (11.72 ± 3.83 vs. 9.00 ± 3.82, *p =* 0.017). In particular, the CT scores for bronchiectasis, nodules, and consolidation were significantly higher in patients with recurrent NTM-PD (*p =* 0.008, *p =* 0.006, and *p =* 0.033, respectively). However, there were no significant differences in cellular bronchiolitis and cavities between the groups (*p =* 0.112 and *p =* 0.784, respectively) (Table 2).

### 3.3. Difference in Treatment Response between Patients with and without NTM-PD Recurrence

The radiologic treatment response was evaluated as the difference between the initial CT score and the CT score at the end of treatment. In patients without NTM-PD recurrence, all the CT scores decreased significantly after treatment completion. However, in patients with NTM-PD recurrence, the bronchiectasis score did not differ significantly between the initiation and termination of the treatment (5.50 ± 1.72 vs. 5.56 ± 1.54, *p* = 0.755). There was no significant difference in the radiologic treatment response between the two groups (Tables S4 and S5).

### 3.4. Reproducibility

Table 3 shows the interobserver agreement between the two readers for scoring of the chest CT findings. Excellent interobserver agreements were observed for the total CT scores (ICC = 0.898, *p <* 0.001), bronchiectasis scores (ICC = 0.876, *p <* 0.001), bronchiolitis scores (ICC = 0.937, *p <* 0.001), cavity scores (ICC = 0.902, *p <* 0.001), and nodule scores (ICC = 0.857, *p <* 0.001). However, the agreement for the consolidation score was fair (ICC = 0.531, *p =* 0.035).

### 3.5. Predictors of Recurrent NTM-PD at the Time of Treatment Termination

**Figure 2:**
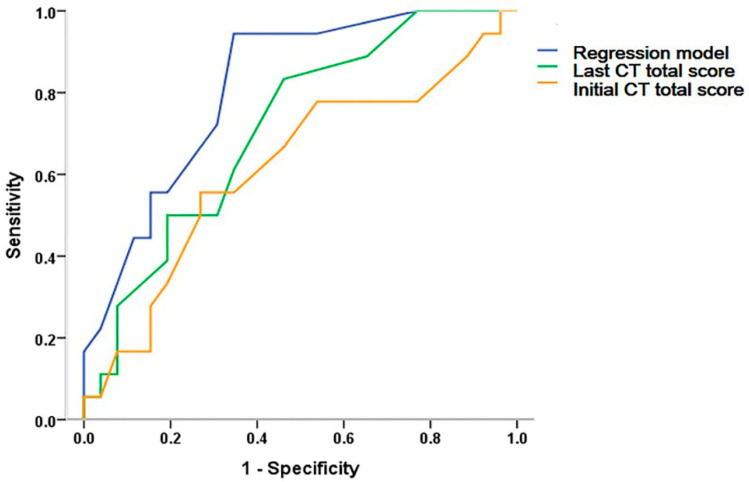
Receiver operating characteristic curve analysis for predicting tuberculous mycobacterial pulmonary disease (NTM-PD). The area under the curve of the total CT score at treatment initiation, treatment termination, and regression model were 0.618 (95% CI = 0.443–0.792), 0.713 (95% CI = 0.561–0.864), and 0.814 (95% CI = 0.689–0.939), respectively.

In univariable logistic regression analysis, initial nodule score, last bronchiectasis score, last nodule score, and last total CT score were significant predictors. Among them, the initial nodule score and the last total score were excluded in multivariable analysis considering multicollinearity. Multivariable regression analysis demonstrated that the scores for bronchiectasis (odds ratio (OR) = 1.638, 95% confidence interval (CI) = 1.049–2.558, *p =* 0.030) and nodules (OR = 5.246, 95% CI = 1.370–20.087, *p =* 0.016) at the time of treatment termination were independent predictors for NTM-PD recurrence (Table 4). In the ROC curve analyses, the AUC value of the total CT score at the time of treatment termination was 0.713 (95% CI = 0.561–0.864). The AUC value of the regression model was 0.814 (95% CI = 0.813–1.000), with a cut-off value of 0.36; the sensitivity and specificity of the model were 82.2% and 69.2%, respectively (Table 4 and Figure 2).

## 4. Discussion

Chest CT scores were analyzed as predictors of recurrence in patients with NTM-PD following successful treatment. Recurrence was observed in 18 (40.9%) of the 44 patients, and the median interval from the treatment termination to recurrence was 578 days (IQR, 333.5–1182 days). The total CT score and scores for bronchiectasis, nodules, and consolidation obtained at the time of treatment termination were significantly higher in patients with recurrent NTM-PD than in those without. In contrast, only CT nodule scores obtained at the time of treatment initiation differed significantly between the two groups. Additionally, the CT scores for bronchiectasis and nodules at treatment termination were acknowledged as independent predictors of disease recurrence, with a regression model AUC of 0.814.

Recurrence after successful treatment of NTM-PD is not rare and has been reported in up to 50% of patients [6,16,17]. The mechanism of disease recurrence is known to be different between NTM-PD and pulmonary tuberculosis. While the persistence of tuberculosis bacilli plays a primary role in the recurrence of pulmonary tuberculosis [18], microbiological recurrence in NTM-PD is predominantly due to reinfection rather than relapse [1,12]. Therefore, NTM-PD is more likely to recur in patients with predisposing factors for the acquisition of new bacterial species, such as structural lung abnormality or compromised immunity.

A previous study reported that the nodular bronchiectatic form on initial chest CT was a significant factor for predicting recurrence after treatment [6,12]. In contrast, our study revealed no significant difference in the initial CT pattern between patients with recurrent and non-recurrent NTM-PD. This inconsistency could be attributed to the different compositions of the study populations; we included a relatively small proportion of patients with fibrocavitary-form NTM-PD in comparison to that in previous studies. Additionally, we included patients with successfully treated NTM-PD regardless of the NTM species; thereby, not confined to MAC. Nevertheless, our study is worthwhile in that we demonstrated the clinical importance of the CT findings obtained at the time of treatment termination rather than treatment initiation. Furthermore, we adopted a detailed CT scoring system and used the score as a predictive radiologic parameter to provide a more objective and accurate assessment of the disease extent and characteristics, instead of a pattern approach.

A previous study showed clinical symptoms as a risk factor of treatment failure in TB patients [19]. However, in our study, the clinical parameters did not differ significantly between the recurrent and non-recurrent groups. In the recurrent group, BMI tended to be low and the treatment duration tended to be prolonged, but there were no significant differences. Instead, CT scores were independent parameters for predicting recurrence. When the cut-off value of the total CT score, obtained at the time of treatment termination, was set to 9.5, sensitivity was 61.1%, and specificity was 65.4% for predicting disease recurrence. Moreover, using the regression model composed of the last bronchiectasis and nodule score (cut-off 0.36) achieved higher performance with a sensitivity and specificity of 82.2% and 69.2%, respectively. The inter-observer agreements for the CT scores were excellent, except for the consolidation score. The reason for the relatively low inter-reader agreement in the consolidation score might be the overlapping of image features between airspace filling (consolidation) and alveolar collapse (atelectasis). Nonetheless, our results suggest that quantitative CT scores can be used as imaging biomarkers for predicting disease recurrence after successful treatment. Thus, in patients showing bronchiectasis and nodules with high CT scores at the time of treatment termination, close monitoring and follow-up are necessary for surveillance of NTM-PD recurrence.

Regarding treatment response, the degree of improvement of each CT score did not differ significantly between the two groups. In both groups, most of the individual parenchymal abnormalities were significantly improved after the treatment, except for the bronchiectasis score in recurrent NTM-PD patients. We may assume that the risk of recurrence could be relatively high when there was a lack of improvement of bronchiectasis and mucus plugging on serial CT scans, and close follow-up would be necessary.

According to Kim et al., peribronchial nodules consequently evolved into inflamed focal cystic bronchiectasis, eventually forming a cavitary lesion in MAC-PD [20]. As nodules and bronchiectasis are involved in the same mechanism of disease progression resulting in structural damage to the lungs, our results showed that both nodule and bronchiectasis scores at the time of treatment termination were predictive of the recurrence of NTM-PD. Our results contribute to bridging gaps in the literature, suggesting that structural lung abnormalities are predisposing host factors for NTM infection and recurrence [21].

Our study has several limitations. First, this was a single-center, retrospective study with a small sample size. Nevertheless, our study is still worthwhile, since we investigated the clinical implication of quantitative CT scores as predictors of NTM-PD recurrence with a long-term follow-up period of up to almost 10 years. Prospective multicentre studies with a larger number of patients are required to confirm our findings. In addition, we did not perform molecular analysis of the NTM species; thus, we were unable to discriminate between relapse (infection with the original strain) and reinfection (infection with a new strain).

We demonstrated that the CT scores at the time of treatment termination had a predictive power for microbiological recurrence following successful treatment of NTM-PD, with good reproducibility. The CT scores of bronchiectasis and nodules were found to be significant determinants for predicting recurrence. We suggest that patients with bronchiectasis and nodules with a high total CT score at the time of treatment termination undergo close monitoring for surveillance of recurrent NTM-PD.

## Figures and Tables

**Table 1:** Baseline characteristics of patients with and without recurrent nontuberculous mycobacterial pulmonary disease.

Characteristics	Non-Recurrent Group (*n* = 26)	Recurrent Group (*n* = 18)	*p*-Value
Age, y *	58.7 ± 11.8	62.3 ± 10.1	0.55
Sex (male: female) ^†^			
Height (m) *	1.61 ± 0.09	1.62 ± 0.07	0.73
Weight (kg) *	51.1 ± 8.1	53.7 ± 8.8	0.18
Body mass index (kg/m^2^) *	19.8 ± 2.6	20.4 ± 2.9	0.12
Pathogen, n (%)			0.74
*M. avium complex*	16 (61.5)	15 (83.4)	
*M. abscessus* subsp. *abscessus*	3 (11.5)	1 (5.5)	
*M. abscessus* subsp. *massiliense*	2 (7.6)	2 (11.1)	
*Others*	3 (11.5)		
Mixed pathogen	2 (7.6)		
Duration of treatment (days) *	585.5 ± 160.8	683.6 ± 123.3	0.17
CT acquisition to treatment initiation (median, day)	21	14.5	0.42
Treatment termination to CT acquisition (median, day)	21.5	28.2	0.61
Initial CT presentation ^†^ n (%)			0.76
Nodular bronchiectatic form	14 (53.8)	11 (61.1)	
Fibrocavitary form	5 (19.2)	2 (11.1)	
Unclassifiable form	7 (26.9)	5 (27.8)	

Values are presented as means ± standard deviations or medians (interquartile ranges). Boldface indicates *p*-values less than 0.05. *p*-values were obtained using the * Mann–Whitney U test and ^†^ Fisher’s exact test. CT, computed tomography.

**Table 2:** Comparison of CT scores between patients with and without recurrent nontuberculous mycobacterial pulmonary disease.

CT Scores	Non-Recurrent Group (*n* = 26)	Recurrent Group (*n* = 18)	*p*-Value
At the time of treatment initiation			
Bronchiectasis (9 points)	4.69 ± 1.72	5.50 ± 1.72	0.119
Cellular bronchiolitis (6 points)	4.35 ± 1.57	4.89 ± 1.49	0.204
Cavity (9 points)	2.96 ± 3.04	2.89 ± 2.85	0.960
Nodules (3 points)	0.58 ± 0.51	1.17 ± 0.86	**0.014**
Consolidation (3 points)	0.69 ± 0.84	0.72 ± 0.83	0.876
Total score (30 points)	13.27 ± 4.21	15.17 ± 4.63	0.187
At the time of treatment termination			
Bronchiectasis (9 points)	4.31 ± 1.62	5.61 ± 1.54	**0.008**
Cellular bronchiolitis (6 points)	2.73 ± 1.46	3.61 ± 1.29	0.112
Cavity (9 points)	1.65 ± 2.45	1.56 ± 2.71	0.784
Nodules (3 points)	0.27 ± 0.45	0.78 ± 0.65	**0.006**
Consolidation (3 points)	0.00	0.17 ± 0.38	**0.033**
Total score (30 points)	9.00 ± 3.82	11.72 ± 3.83	**0.017**

Values are presented as means ± standard deviation. Boldface indicates *p*-values less than 0.05. *p*-values were obtained using Mann-Whitney U test. CT, computed tomography.

**Table 3:** Interobserver agreement for computed tomography (CT) scoring.

CT Scores	ICC	95% CI	*p*-Value
Bronchiectasis (9 points)	0.876	0.729–0.944	<0.001
Cellular bronchiolitis (6 points)	0.937	0.861–0.971	<0.001
Cavity (9 points)	0.902	0.785–0.955	<0.001
Nodules (3 points)	0.857	0.687–0.935	<0.001
Consolidation (3 points)	0.531	0.063–0.794	0.035
Total score (30 points)	0.841	0.651–0.928	<0.001

ICC, intraclass correlation coefficient; CI, confidence interval.

**Table 4:** Results of logistic regression analysis and predictor variables.

	Univariable Regression		Multivariable Regression		
Variables	Odds Ratio (95% CI)	*p*-Value	Odds Ratio (95% CI)	*p*-Value	Beta Coefficient
Initial nodule score	4.344 (1.255, 15.038)	0.020			
Last nodule score	5.268 (1.543, 17.983)	0.008	5.246 (1.370–20.087)	0.016	1.657
Last bronchiectasis score	1.653 (1.075, 2.541)	0.022	1.638 (1.049–2.558)	0.030	0.494
Last total score	1.209 (1.012, 1.443)	0.036			
Model constant				0.004	−3.627

The logistic regression equation is log_e_ (Odds of recurrence) = −3.627 + 1.638 (CT bronchiectasis score) + 5.246 (CT nodules score). CI, confidential interval.

## Data Availability

Data available on request due to restriction. The data presented in this study are available on request from the corresponding aurthor. The data are not publicly available due to ethics.

## Supplementary Materials

The following are available online at https://www.mdpi.com/2077-0383/10/2/172/s1, Table S1: CT scoring system for the assessment of the extent of nontuberculous mycobacterial pulmonary disease, Table S2: Changes in isolated species in recurrent NTM-PD, Table S3: Treatment regimen of NTM-PD in 44 patients, Table S4: Comparison of the difference in CT score obtained at the treatment initiation and treatment termination between recurrent and non-recurrent patients, Table S5: Comparison of CT scores obtained at the treatment initiation and at the treatment termination in patients without recurrent NTM-PD (a) and with recurrent NTM-PD (b).
