# Supplementary material for: High-Resolution CT Findings as Predictive Factors for Recurrent Nontuberculous Mycobacterial Pulmonary Disease after Successful Treatment
Source: J Clin Med. 2021 Jan 6;10(2):172. doi: 10.3390/jcm10020172 (PMC7825014; doi:10.3390/jcm10020172)
Supplement: Supplementary file 1 [file jcm-10-00172-s001.pdf]

Electronic Supplementary Materials

Table S1. CT scoring system for the assessment of the extent of nontuberculous mycobacterial pulmonary disease

CT findings	Score 0	Score 1	Score 2	Score 3
Bronchiectasis (9 points)				
Severity*	Absent	Mild	Moderate	Severe
Extent†	Absent	1–5	6–9	> 9
Mucus plugging†	Absent	1–5	6–9	> 9
Cellular bronchiolitis (6 points)				
Severity‡	Absent	Mild	Moderate	Severe
Extent†	Absent	1–5	6–9	> 9
Cavity (9 points)				
Diameter (cm)	Absent	< 3	3–5	> 5
Wall thickness (mm)	Absent	< 1	1–5	> 5
Extent§	Absent	1–3	4–5	> 5
Nodules (3 points) †	Absent	1–5	6–9	> 9
Consolidation (3 points) †	Absent	< 3	3–5	> 5

*Mild = bronchus diameter greater than the adjacent vessel diameter; moderate = bronchus diameter two to three times greater than the vessel diameter; severe = bronchus diameter three times greater than the vessel diameter

†Data are presented as the number of segments.

‡Mild = identifiable, peripheral lung < 1 cm from the pleura; moderate = definite, involvement greater than 1–3 cm from the pleura; severe = extensive, extension to the central lung

§Data are presented as the number of cavities.

CT, computed tomography

Table S2. Changes in isolated species in recurrent NTM-PD

Initial isolated species	Recurred isolated species	Number of patients
M. avium (n=9)	M. avium	2
	M. intracellulare	4
	M. abscessus	1
	Mixed	2
M. intracellulare (n=7)	M. avium	1
	M. intracellulare	3
	M. abscessus	3
M. abscessus (n=1)	M. abscessus	1
M. massiliense (n=2)*	M. massiliense	1
	Not available	1

Table S3. Treatment regimen of NTM-PD in 44 patients

Characteristics	Non-recurrent group (n = 26)	Recurrent group (n = 18)
M. avium complex (n = 31)		
Macrolide + rifampin + ethambutol	15	13
Macrolide + ethambutol	1	1
Macrolide + rifampin + moxifloxacin	0	1
M. abscessus complex (n = 8)		
Macrolide + cefoxitin + amikacin	3	3
Macrolide + imipenem + amikacin	2	0
M. kansasii (n = 2)		
Isoniazid + rifampin + ethambutol	2	0
M. xenopi (n = 1)		
Macrolide + rifampin + ethambutol	1	0
Mixed pathogen (n =2)		
Macrolide + cefoxitin + amikacin	1	0
Macrolide + cefoxitin + amikacin + rifampin + ethambutol	1	0

Table S4. Comparison of the difference in CT score obtained at the treatment initiation and at the treatment termination between recurrent and non-recurrent group

Differences in CT scores (CT $Score_{initiation}$- CT $Score_{termination}$)	Non-recurrent group (n=26)	Recurrent group (n=18)	p-value*
Bronchiectasis (9 points)	0.38 ± 0.80	-0.06 ± 0.94	0.176
Cellular bronchiolitis (6 points)	1.62 ± 1.39	1.28 ± 1.13	0.521
Cavity (9 points)	1.31 ± 1.64	1.33 ± 1.94	0.717
Nodules (3 points)	0.31 ± 0.55	0.39 ± 0.50	0.663
Consolidation (3 points)	0.69 ± 0.84	0.56 ± 0.71	0.653
Total score (30 points)	4.27 ± 2.78	3.39 ± 3.36	0.442

Data are mean value ± standard deviation P-values were obtained using the *Mann-Whitney test.
CT, computed tomography

Table S5. Comparison of CT score obtained at the treatment initiation and at the treatment termination in patient without recurrent NTM-PD (a) and with recurrent NTM-PD (b).

(a) Patients without NTM-PD recurrence (n=26)

CT scores	At the time of treatment initiation	At the time of treatment termination	p-value*
Bronchiectasis (9 points)	4.69 ± 1.72	4.31 ± 1.62	**0.025**
Cellular bronchiolitis (6 points)	4.35 ± 1.57	2.73 ± 1.46	**< 0.001**
Cavity (9 points)	2.96 ± 3.04	1.65 ± 2.45	**0.001**
Nodules (3 points)	0.58 ± 0.50	0.27 ± 0.45	**0.011**
Consolidation (3 points)	0.69 ± 0.84	0.04 ± 0.20	**< 0.001**
Total score (30 points)	13.27 ± 4.21	9.00 ± 3.82	**< 0.001**

Data are mean value ± standard deviation. Boldface indicates p-values less than 0.05. P-values were obtained using the * Wilcoxon rank sum test. CT, computed tomography

(b) Patients with NTM-PD recurrence (n=18)

CT scores	At the time of treatment initiation	At the time of treatment termination	p-value*
Bronchiectasis (9 points)	5.50 ± 1.72	5.56 ± 1.54	0.755
Cellular bronchiolitis (6 points)	4.89 ± 1.49	3.61 ± 1.29	**0.002**
Cavity (9 points)	2.89 ± 2.85	1.56 ± 2.71	**0.017**
Nodules (3 points)	1.17 ± 0.86	0.78 ± 0.65	**0.008**
Consolidation (3 points)	0.72 ± 0.83	0.17 ± 0.38	**0.004**
Total score (30 points)	15.17 ± 4.63	11.72 ± 3.83	**< 0.001**

*Data are mean value ± standard deviation. Boldface indicates p-values less than 0.05. P-values were obtained using the *Wilcoxon rank sum test. CT, computed tomography
